# Duodenal Pyloric Gland Adenoma in a 59-Year-Old Asian Male

**DOI:** 10.1155/2018/9287843

**Published:** 2018-06-04

**Authors:** Mia Manabat, Matthew Jackson, Kha Ngo, Laurence Stawick

**Affiliations:** ^1^St John Providence Hospital, USA; ^2^St John Macomb-Oakland Hospital, USA

## Abstract

Pyloric gland adenomas (PGAs) are rare precancerous tumors typically arising from the stomach. Even more rarely do they arise in extragastric sites such as the duodenum and gallbladder. The identification of PGAs is important because they possess a risk of developing into invasive adenocarcinoma. This case report describes a 59-year-old male who presented to our office for a follow-up of a nonspecific duodenal lesion. Endoscopic ultrasound and pathology revealed a PGA with low grade dysplasia and some areas of focal high grade dysplasia. It is important that gastroenterologists are aware of these lesions and their risk of malignant transformation. More studies are needed to describe the long-term behavior of pyloric gland adenomas and to recommend appropriate endoscopic surveillance of these lesions.

## 1. Introduction

Pyloric gland adenomas (PGAs) are rare precancerous tumors typically arising from the stomach. They account for about 2.7% of all gastric polyps. Even more rarely do they arise in extragastric sites such as the esophagus, gallbladder, bile duct, duodenal bulb, and duodenum. These lesions are most common in females in their 8th decade of life [1, 2] likely due to their association with autoimmune gastritis. PGAs were originally defined in 1976 by Kurt Elster [3] and there is speculation that these lesions are often underrecognized. The identification of these lesions is important because they possess a risk of developing into invasive adenocarcinoma. In past studies, the transition of PGAs to adenocarcinoma is noted to be as high as 28% [1]. As of yet, there are no long-term studies following these lesions and there are no recommendations regarding the need for long-term surveillance.

## 2. Case Report

**Figure 1:**
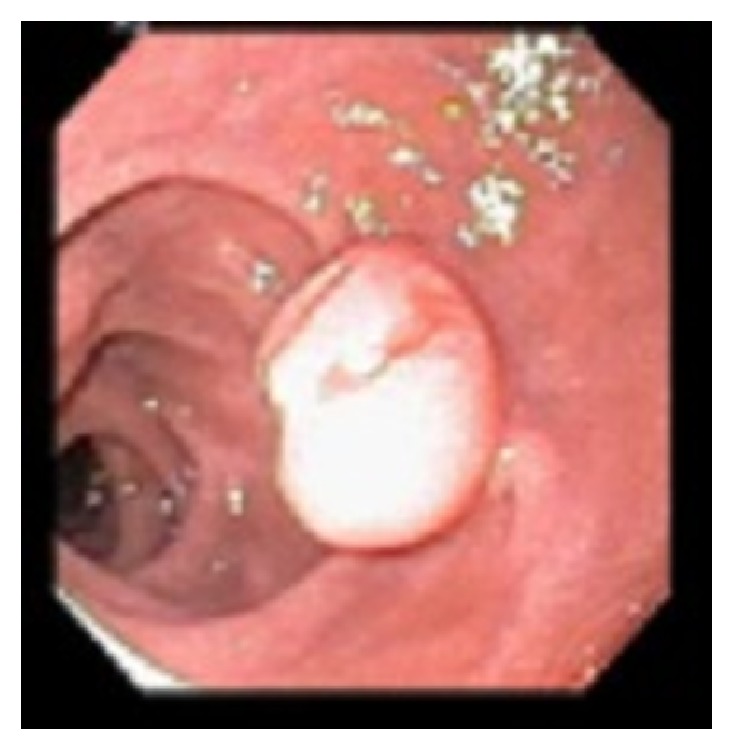
PGA located in the second portion of the duodenum opposite to the ampulla.

**Figure 2:**
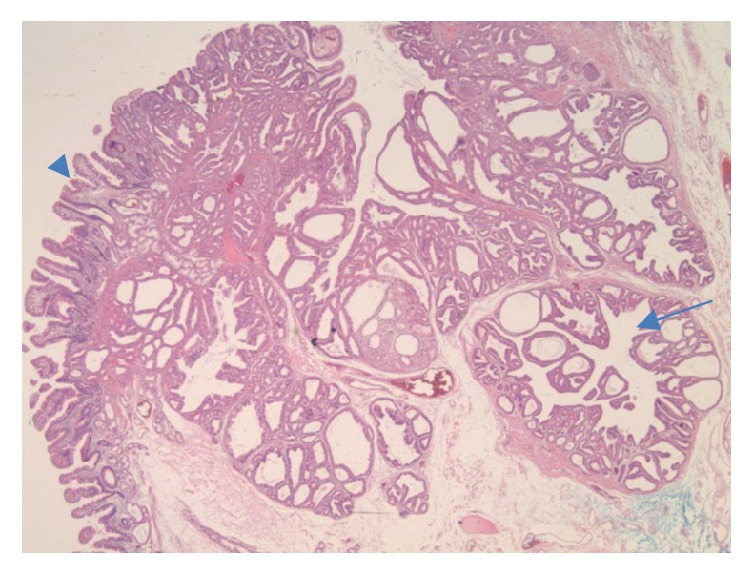
Low power view of the PGA (arrow) arising from the duodenum with normal villous mucosa (arrowhead).

**Figure 3:**
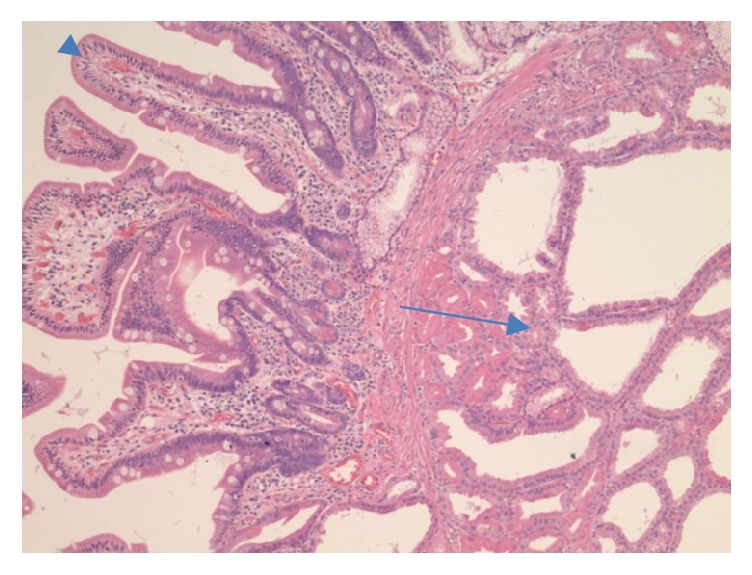
High power view of the PGA (arrow) arising from the duodenum with normal villous mucosa (arrowhead).

**Figure 4:**
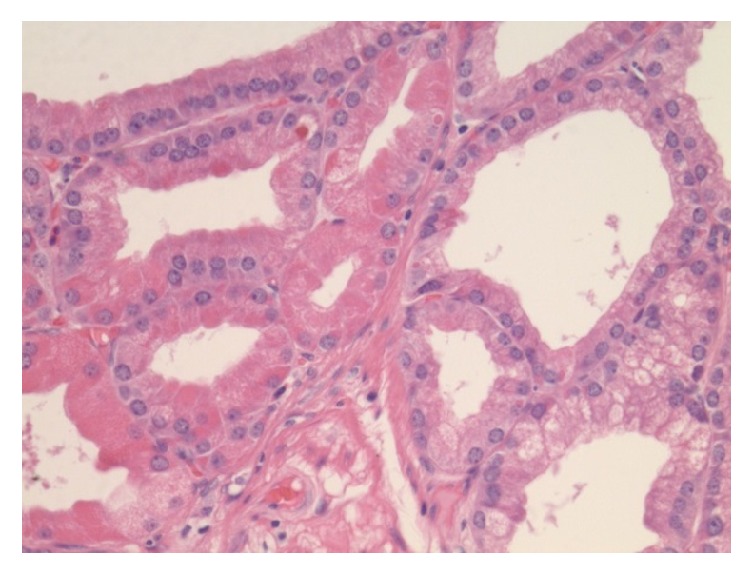
High power view of the PGA showing the ground glass appearance of cytoplasm and a monolayer of round nuclei.

**Figure 5:**
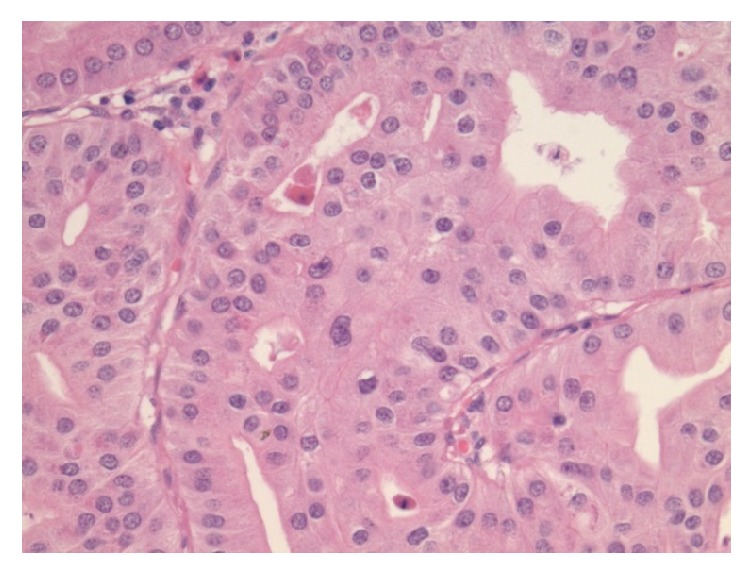
High power view exhibiting small amount of atypia of the PGA.

**Figure 6:**
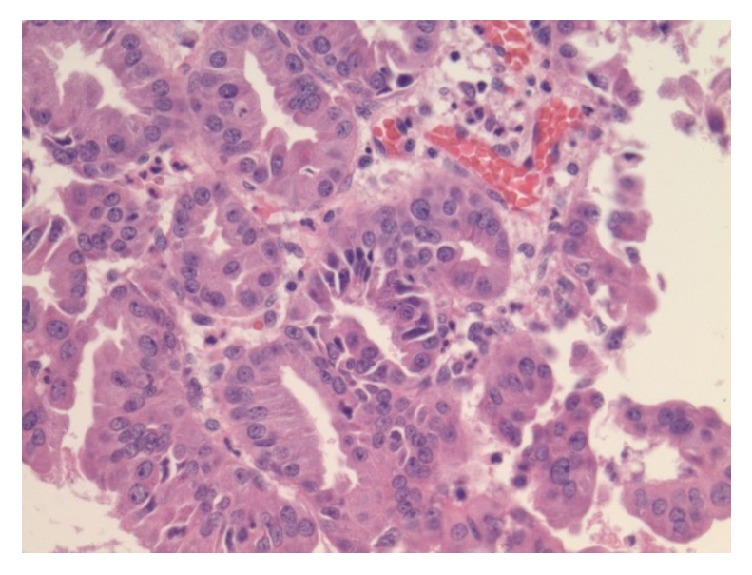
High power view exhibiting cell atypia including nuclear membrane irregularity and nuclear pleomorphism.

A 59-year-old Asian male presented to the gastroenterology office for a follow-up of a previously found duodenal lesion. He had an EGD at an outside facility 2 years earlier for evaluation of abdominal pain which showed a 1.2 cm duodenal polypoid lesion in the second portion of the duodenum. Pathology of the polyp revealed heterotopic gastric mucosa with benign lobules of gastric glands. He also was found to have chronically active H. pylori gastritis with numerous Helicobacter organisms and atrophic gastritis with patchy areas of intestinal metaplasia. At that time, he was instructed to follow up for a repeat endoscopy for surveillance in two years. As recommended, two years later he presented to our office for surveillance of his duodenal lesion. Due to his history of atrophic gastritis and a large duodenal lesion, we performed an upper endoscopy that showed atrophic gastritis, duodenitis, and a single 10 mm polyp in the second portion of the duodenum. The duodenal polyp was biopsied. Pathology revealed polypoid gastric metaplastic mucosa with focal epithelial atypia including nuclear enlargement, stratification, nucleoli, and few mitoses. The patient was referred for endoscopic ultrasound to further characterize the lesion and complete resection. Several weeks later he underwent an endoscopic ultrasound that revealed a 13-mm pedunculated and sessile polyp on the lateral wall opposite to the major papilla in the second portion of the duodenum (Figure 1). The polyp was removed* en bloc* using a hot snare following a 4-mL saline lift. Two endoclips were placed for hemostasis. Pathology revealed a pyloric gland adenoma (Figures 2, 3, and 4) with predominantly low grade dysplasia (Figures 5 and 6) and some small areas of focal high grade dysplasia. The slides were read by the in-house pathologist and were also reviewed by a GI expert pathologist at the University of Michigan. The patient was recommended to follow up for surveillance upper endoscopy in 6 months due to the areas of high grade dysplasia found on pathology. He was lost to follow-up for over one year. Fifteen months after the PGA was removed, the patient did follow-up for a surveillance upper endoscopy. A duodenal scar was found at the site of the PGA and a biopsy was taken from the site. There was no evidence of residual pyloric gland adenoma. The patient was then recommended to follow up for surveillance endoscopy in 5 years in accordance with the ASGE guidelines for surveillance of gastric adenomatous polyps.

## 3. Discussion

Pyloric gland adenomas are rare neoplasms of the gastrointestinal tract. They are typically found in the stomach but can also originate from the duodenum, gallbladder, bile duct, pancreas, rectum, and heterotopic gastric mucosa in Barrett's esophagus. They have also been reported in the cervix. According to the Vieth study in 2003 in a registry of 2778 gastric polyps diagnosed at the Institute of Pathology of Bayreuth between 1990 and 2000, only 2.7% were found to be pyloric gland adenomas. In the same study, 61% of the adenomas were found in females and the average age for females was 75 [1]. Only 1 case in this series of 90 patients was found to have a duodenal PGA and 7 cases were found in the duodenal bulb (8.8%). Contrarily, a study done by Chen in 2009 found 19 of 41 PGAs were found in the duodenum (46.4%). Of these 19 duodenal PGAs, there was no statistical difference in males versus females. Of note, there was a statistical preference towards females in PGAs arising in the stomach [2]. Although these lesions are generally rare it appears that there has been a significant increase in the number of PGAs diagnosed. The cause for the increasing incidence is unknown; however it is possible that PGAs have been underdiagnosed for many years. The gross or endoscopic appearance of PGAs has been described as dome-shaped or polypoid. We described the lesion in this case as a pedunculated and polypoid lesion and measured it to be 1.3 cm. The reported average size at diagnosis is 1.6 cm [1].

Pyloric gland adenomas have also been reported in association with autoimmune gastritis in 33.9% of patients, active* H. pylori* associated gastritis in 30.2%, and only 3.8% of those with normal gastric mucosa [1]. Similarly, Chen et al. found a 40% association of gastric PGAs with autoimmune gastritis. This could potentially pose an interesting basis for further studies of PGAs. It may also explain why there is a significant female predominance, particularly in gastric PGAs, considering autoimmune diseases are much more prevalent in females.

According to the Chen study performed in the US between 2004 and 2008, 36% of patients diagnosed with PGA had no dysplasia, 12% had low grade dysplasia, 39% had high grade dysplasia, and 12% had invasive carcinoma. In the Vieth study performed in Germany between 1990 and 2000, 30% of pyloric gland adenomas were found to have invasive carcinoma. Although there is a discrepancy in the rate of transformation into carcinoma, it has been well-established that there is a significant risk of becoming cancerous. There may be a discrepancy due to the expertise in pathologists diagnosing these lesions. PGAs were first described in Europe and have recently become more recognized in the US. There are currently no specific guidelines for the removal and surveillance of pyloric gland adenomas. However the ASGE GI Endoscopy Guidelines from April 2006 recommend that adenomatous gastric polyps be resected and surveillance endoscopy be performed at 3-5-year intervals [4]. It is important that gastroenterologists are aware of these lesions and their risk of malignant transformation. More studies are needed to describe the long-term behavior of pyloric gland adenomas and the utility and cost-effectiveness of surveillance endoscopy of these lesions.
